# Acceptance, Awareness, and Knowledge of Human Papillomavirus Vaccine in Eastern Province, Saudi Arabia

**DOI:** 10.7759/cureus.31809

**Published:** 2022-11-22

**Authors:** Abdullatif K Almaghlouth, Abdullah H Bohamad, Roaa Y Alabbad, Jehad H Alghanim, Danah J Alqattan, Reda A Alkhalaf

**Affiliations:** 1 Urology, King Faisal University Hospital, Hofuf, SAU; 2 Infectious Disease, King Faisal University, Hofuf, SAU

**Keywords:** hpv infection, hpv vaccines, malignancy, human papillomavirus (hpv), vaccine, infection

## Abstract

Introduction: Human papillomavirus (HPV) is a virus infection and can lead to different epithelial lesions and cancer. Nevertheless, HPV infection is a disease that can be prevented with vaccine. Genital warts, cancer, and HPV infections can all be prevented with this vaccine. In this study, we aimed to investigate awareness, knowledge of HPV infection, and acceptance of its vaccine among the general population in Saudi Arabia.

Methodology: An observational cross-sectional study was conducted in Eastern Province, Saudi Arabia between September and October 2022, using a self-administered validated questionnaire. beginning with informed consent, followed by questions about demographic data. Finally, there were questions about participants’ knowledge, awareness, and attitudes around HPV and its vaccine.

Rustles: The results showed 645 participants and only 4% of them had received their HPV vaccination. Knowledge, awareness, and attitude levels were all 35% with mean of 1.14, 28% with mean of 3.03, and 51% with mean of 2.02, respectively. Social media was found to be the most reliable source of information about the HPV vaccine (33%), followed by health practitioners (21%). The most common reasons for refusing vaccinations are “belief we are healthy” (48%), followed by “lack of information” (38%).

Conclusion: Regarding HPV infection and its vaccination, Saudi Arabia significantly lacks knowledge and awareness. It is essential to provide instructions and information in schools for students, families, and healthcare providers about infection and its vaccination.

## Introduction

Human papillomavirus (HPV) is a sexually transmitted infection that can cause various epithelial lesions and malignancies. It has been linked to cervical, penial, oropharynx, and lung cancer. HPV subtypes 16 and 18 carry a high substantial risk of causing cervical and penial malignancy [1]. In 2020, it was estimated that there were 604,000 new cases and 342 deaths worldwide of cervical and 36,068 new cases and 13,211 deaths of penile cancer. Cervical cancer ranks as the fourth most prevalent cancer type in women globally [2,3]. In 2020, Saudi Arabia reported 358 new cases of cervical cancer and 179 deaths due to its complications, and four new cases and two deaths of penile cancer. Moreover, cervical cancer ranks eighth and penile ranks as 35th most common malignancy in Saudi Arabia [4]. Fortunately, HPV infection is considered as a preventable disease by using vaccination. This vaccine is available in Saudi Arabia for free. The HPV vaccine can decrease HPV 6, 11, 16, 18 infections and genital warts by up to 90% and can decrease high-grade cervical lesions by up to 85% [5]. The World Health Organization (WHO) recommends that females and males between the age of 9 and 14 years receive a two-dose regimen of the HPV vaccine and females aged 13 to 26 years should also obtain catch-up three doses of the vaccine [6]. A considerable number of studies have been published on knowledge, awareness, and acceptance regarding the HPV vaccine. We found a study conducted among Arab countries that reported poor awareness and knowledge of HPV [7]. In Saudi Arabia, there is poor awareness and knowledge of HPV, its vaccine, and cervical cancer among females [8]. To our knowledge, there is only one study in Saudi Arabia to assess the awareness knowledge of HPV and acceptance of its vaccine. Our aim of the study is to assess awareness, knowledge of HPV, and acceptance of its vaccine.

## Materials and methods

Study design and selection criteria

We conducted an observational cross-sectional study using a self-administered validated questionnaire to obtain responses from the male and female citizens of Eastern Province, Saudi Arabia who were older than 12 years old, and willing to participate in the study. Non-Saudi citizens, not living in Eastern Province, Saudi Arabia who were younger than 12 years old, and anyone who refused to participate or had not completed the entire questionnaire were excluded. The questionnaire was distributed randomly as Google form using social media platforms. WhatsApp, Telegram, and Instagram applications were utilized to collect different groups and people.

Questionnaire design and validity

We did an extensive literature review before writing the questionnaire. After that, we wrote our questionnaire and presented it to three experts to get face validity. After getting face validity, we conducted a pilot study on 20 participants who were excluded from the final rustle. Our questionnaire was designed in Arabic, as it is the native language of Saudi Arabia. It had three sections and was developed and published between September 2022 and October 2022. The questionnaire took approximately three minutes to be completed. The first section started with informed consent from participants. Then the second section was designed to obtain information about the demographical data such as age, gender, marital status, educational level, and income. The third section included questions regarding the participants’ knowledge, awareness, and attitude toward HPV.

Statistical analysis

Descriptive analysis and Chi-square test were used to analyze the data from the questionnaire, with a significance level of 0.05. The sample size was 645; with a confidence level = 95%, and an acceptable error = 5%. The target population = 34.81 million. This study was approved by the Ethics Committee of King Faisal University (Ethical approval code KFU-REC-2022-SEP-ETHICS92). Before filling out the survey, and according to the Helsinki declaration, there was a statement that declared that participants’ confidentiality and privacy were guaranteed. After data was extracted, it was revised, coded, and entered into the statistical software IBM Statistical Package for the Social Sciences (SPSS) version 22 (SPSS, Inc. Chicago, IL).

## Results

Demographic characteristics of participants

The results showed, there were 644 participants; 64% females and 36% males. Age ranged from 12 to 60 years with a mean of 28.82 years, 38 participants were younger than 18 years and 606 were older than 18 to 60 years, and a standard deviation of 11.35. 4.8% only knew someone with cervical cancer and only 2.20% had a Pap smear in the last 3 years while 0.50% had it more than 3 years ago (Table 1).

**Table 1:** Demographic data

Variables	Categories			Count	%
Gender	Male			232	36.00%
	Female			412	64.00%
Marital status	Single			361	56.10%
	Married			267	41.50%
	Divorced			12	1.90%
	Widowed			4	0.60%
Educational level	Secondary			170	26.40%
	Diploma			39	6.10%
	Bachelor			376	58.40%
	Master			16	2.50%
	Less than high school			27	4.20%
	Primary			11	1.70%
	Doctoral			5	0.80%
Occupational status	Governmental sector			189	29.30%
	Private sector			75	11.60%
	Unemployed			345	53.60%
	Retired			17	2.60%
	Self-employed			18	2.80%
Monthly family income	Less than 3000 RS/ month			88	13.70%
	3000-10000 RS/month			203	31.50%
	10,000-20,000 RS/ month			243	37.70%
	More than 20,000 RS/month			110	17.10%
Age	Min	Max	Mean	St. Deviation	
	12	60	28.82	11.35	
Do know someone with cervical cancer?	Yes			31	4.80%
	No			613	95.20%
Pap smear in the last 3 years	Yes			9	2.20%
	Yes, more than 3 years ago			2	0.50%
	No			391	97.30%

Knowledge and awareness of participants about HPV

Knowledge level was 35% with a mean of 1.41. Regarding the order of the items, the most common was “HPV causes cervical cancer” (65%), then “HPV causes penis cancer” (36%), then “HPV is a virus that is sexually transmitted” (30%), and finally “HPV will usually go away on its own without treatment” (11%) (Table 2). The most common source of information about the HPV vaccine was social media (33%), followed by health practitioners (21%) (Table 3). The awareness level was 28% with a mean of 3.03. Table 4 provides information on whether the provider explains the benefits and risks of vaccination. Regarding the order of the items, the most common was “Does the HPV vaccine be effective against HPV?” (46.4%), then “Have you ever heard about HPV?”(42.2%). The least was “Do females need to be screened for HPV before vaccinated”(8.4%), followed by “Do males need to be screened for HPV?” (Table 5).

**Table 2:** Knowledge level

Items		Yes	No	I don’t know	True answer
HPV is a virus that is sexually transmitted.	Count	191	50	403	191
	%	29.70%	7.80%	62.60%	30%
HPV will usually go away on its own without treatment.	Count	68	162	414	68
	%	10.60%	25.20%	64.30%	11%
HPV causes penis cancer?	Count	234	247	163	234
	%	36.30%	38.40%	25.30%	36%
HPV causes cervical cancer?	Count	418	105	121	418
	%	64.90%	16.30%	18.80%	65%
Mean score	1.41(35%)				

**Table 3:** Sources of information about HPV vaccine

Source	Count	Percent
Social media	112	33%
Health practitioners	69	21%
My medical study	4	1%
Governmental official data	57	17%
General health promotion	42	13%
Friends, relatives, or family	51	15%

**Table 4:** The provider explains the benefits and risks of vaccination

Item		No	Yes
If your answer was yes, did the provider explain the benefits and risks of vaccination	Count	3	23
	%	11.50%	88.50%

**Table 5:** Awareness level

Items		Yes	No	I don't know		True answer
Have you ever heard about HPV?	Count	272	372			272
	%	42.20%	57.80%			42.4%
Have you ever heard about HPV vaccine?	Count	227	417			227
	%	35.2	64.8			35.2%
Does the HPV vaccine be effective against HPV?	Count	299	28	317		299
	%	46.40%	4.30%	49.20%		46.4%
Does HPV vaccine protect against some types of cancer?	Count	249	62	333		249
	%	38.70%	9.60%	51.70%		38.7%
Is HPV vaccine available in your country?	Count	251	13	380		251
	%	39.00%	2.00%	59.00%		39%
Can the HPV vaccine cause side effects?	Count	117	86	441		86
	%	18.20%	13.40%	68.50%		13,4%
Can the HPV vaccine cause HPV infection?	Count	73	164	407		164
	%	11.30%	25.50%	63.20%		25.5%
Does the HPV vaccine decrease the chance of having changes in the Pap smear?	Count	159	50	435		159
	%	24.70%	7.80%	67.50%		24.7%
Do females need to be screened for HPV before being vaccinated?	Count	257	54	333		54
	%	39.90%	8.40%	51.70%		8.4%
Do males need to be screened for HPV before being vaccinated?	Count	169	90	385		90
	%	26.20%	14.00%	59.80%		14%
		From 11-26 years	From 27-45 years	Both	I don't know	
Who should be vaccinated with HPV vaccine?	Count	122	103	146	273	103
	%	18.90%	16.00%	22.70%	42.40%	16%
Mean score	3.03(28%)					

Acceptance and attitude of participants about HPV vaccine

The attitude level was 51% with a mean of 2.02. 4% only of the participants got their vaccinations for HPV. There were 23 (88.5%) participants who knew the benefits and risks of vaccination, and three (11.5%) did not. 47.7% were willing to receive the HPV vaccine which can protect against HPV infection while 52.3% weren’t. There were 17.7% who would recommend the HPV vaccine for a child or adolescent (age between 9 and 12 years old), and 14.8% wouldn’t. 67.3% could not decide according to their information. 28.3% would recommend the HPV vaccine for a friend or relative, 6.2% wouldn’t, and 65.5% couldn’t decide according to their information (Table 6). The most common reasons for not willing to be vaccinated were the belief we are well (48%), then lack of information (38%), then considering screening too risky (8%) then inability to afford and thinking it is not beneficial (3%) (Table 7). Knowledge level was 35%, awareness level was 28%, and attitude level was 51% (Table 8). Knowledge, awareness, and attitude information about HPV according to demographic data is presented in Table 9.

**Table 6:** Attitude level

Items		Yes	No	
Did you get vaccination for HPV?	Count	26	618	
	%	4.00%	96.00%	
Are you willing to receive the HPV vaccine which can protect against HPV infection?	Count	307	337	
	%	47.70%	52.30%	
		Yes	No	Cannot decide according to my information
Would you recommend the HPV vaccine for a child or adolescent (age between 9 and 12 years old)?	Count	114	95	435
	%	17.70%	14.80%	67.50%
Would you recommend the HPV vaccine for a friend or relative?	Count	182	40	422
	%	28.30%	6.20%	65.50%
Mean attitude	2.02 (51%)			

**Table 7:** Reasons for not willing to be vaccinated

Reasons	Frequencies	Percent
Belief we are well	199	48%
Lack of information	157	38%
Consider screening too risky	34	8%
Inability to afford	13	3%
Thinking it is not beneficial	14	3%

**Table 8:** Knowledge, awareness, and attitude about HPV

Level	Mean	Percent
Knowledge	1.41	35%
Awareness	3.03	28%
Attitude	2.02	51%

**Table 9:** Knowledge, awareness, and attitude about HPV according to demographic data

Variables	Categories	Knowledge	Awareness	Attitude
Sex	Male	1.33	2.84	2.13
	Female	1.46	3.14	1.97
	Statistics	-1.319	-1.356	2.481
	p-value	0.188	0.176	0.014
Marital status	Single	1.39	3.00	2.01
	Married	1.46	3.12	2.06
	Divorced	1.25	2.33	1.67
	Widowed	0.75	2.00	1.75
	Statistics	0.658	0.595	1.248
	p-value	0.578	0.618	0.292
Educational level	Secondary	1.39	2.82	2.09
	Diploma	1.08	2.62	1.97
	Bachelor	1.47	3.21	1.98
	Master	1.94	3.50	2.25
	Less than high school	1.15	2.19	2.22
	Primary	0.73	2.00	1.82
	Doctoral	2.40	5.80	2.20
	Statistics	2.591	2.350	1.083
	p-value	0.30	0.017	0.371
Occupational status	Governmental sector	1.48	3.30	2.17
	Private sector	1.36	2.73	1.91
	Unemployed	1.39	2.98	1.97
	Retired	1.35	2.76	1.94
	Self-employed	1.61	2.78	2.06
	Statistics	0.347	0.815	2.634
	p-value	0.846	0.516	0.033
Monthly family income	Less than 3000 RS/ month	1.18	2.35	2.00
	3000-10000 RS/month	1.42	3.10	2.00
	10,000-20,000 RS/ month	1.54	3.25	2.13
	More than 20,000 RS/month	1.31	2.98	1.85
	Statistics	2.352	2.536	3.357
	p-value	0.071	0.056	0.019
Age	Statistics	-0.011	0.029	0.070
	p-value	0.775	0.461	0.078

## Discussion

HPV is a sexually transmitted infection that can cause various epithelial warts and malignancies, it has been linked to many types of cancer [9,10]. This study investigated the level of awareness knowledge, and acceptance of HPV infection and its vaccine among the general population in the Eastern Province of Saudi Arabia. Our finding showed a low level of acceptance and attitudes among participants regarding the HPV vaccine. Most of the participants had a low level of awareness and knowledge regarding HPV infection and its vaccine.

Awareness and knowledge level

In our study, the result showed that the awareness level of HPV infection and vaccine was significantly low which explains why only 4% of participants had received the vaccination. Almost one-third of the participants believed that HPV can be transmitted by sexual activity, 65% agreed that cervical cancer could be caused by HPV, and almost one-third of participants believed penial cancer could be caused by HPV. The present findings seem to be consistent with a study conducted in India showed that knowledge and awareness levels were poor regarding HPV and its vaccine and there was a statistically significant difference between the awareness level and educational level and age [11]. Another study in Europe showed similar findings regarding knowledge about HPV and its vaccine [12]. Also, one study conducted in sub-Saharan Africa including several countries showed a lack of knowledge and moderate awareness level regarding cervical cancer, HPV, and its vaccine [13].

Attitude and acceptance

Attitude and acceptance levels were significantly low obviously among females and with low education levels. The findings of the current study are consistent with two studies conducted in Saudi Arabia. The first study conducted in the capital of Saudi Arabia, Riyadh, reported a significant low attitude and acceptance level which was the lowest rate among other studies [8]. The second one in Jazan showed similar findings regarding attitude and acceptance level with our study [14]. These studies reflected low attitude and acceptance levels among Saudis. Our findings showed almost half of the participants believed that the most common reason for not willing to be vaccinated is that they believed they are well (48%), and the second common reason was lack of information (38%). These findings of our study are not consistent with the Jazan study which showed the most common reasons for not willing to be vaccinated were considering this vaccine new 38% of individuals thought the vaccination was too new, and a third of participants were concerned that it might promote earlier sexual behavior. and about 37% were worried about the HPV vaccine’s effectiveness, while 45% were worried about vaccine safety [14].

Limitations and recommendation

The limitations of our study are that gender differences were not evaluated and not covered in large demographic areas in Saudi Arabia. We were not able to include Saudi citizens who live in other countries. A future study should include a large area in different regions and include Saudi citizens who live abroad and compare and assess knowledge, awareness, and acceptance with them.

## Conclusions

Our study showed a significant lack of knowledge and awareness regarding HPV infection and vaccines. Providing education and knowledge in schools for students and family, making campaigns, and using social media for education are important to increase the level of awareness and attitude. Additionally, the Ministry of Health should print educational materials and make them available in public and governmental organizations. Increasing levels of knowledge, awareness of screening, and early detection of malignancy are crucial.
